# 非小细胞肺癌T790M基因突变研究进展

**DOI:** 10.3779/j.issn.1009-3419.2013.06.08

**Published:** 2013-06-20

**Authors:** 慧 李

**Affiliations:** 130012 长春，吉林省肿瘤医院胸部肿瘤内一科 Department of Thoracic Oncology, Jilin Provincial Cancer Hospital, Changchun 130012, China

**Keywords:** 肺肿瘤, 表皮生长因子受体, T790M, 酪氨酸激酶抑制剂, Lung neoplasms, EGFR, T790M, TKI

## Abstract

携带表皮生长因子受体（epidermal growth factor receptor, *EGFR*）基因活性突变的非小细胞肺癌（non-small cell lung cancer, NSCLC）晚期患者使用EGFR-受体酪氨酸激酶抑制剂（tyrosine kinase inhibitor, TKI）治疗后具有较好的临床获益，但大部分患者在使用该药治疗10个月后出现耐药现象。研究发现EGFR基因20号外显子T790M基因突变是导致EGFR-TKI耐药的最主要因素，但其作用机制至今未明。目前的研究结果显示T790M基因突变是一个独立的、好的预后因素，但其能否作为EGFR-TKI的疗效预测因子仍存在争议。近年来，针对NSCLC肿瘤中T790M基因突变的检测技术不断更新，针对T790M耐药的新的治疗策略也不断涌现。本文就NSCLC中T790M基因突变的耐药机制、临床意义、检测方法及应对策略等方面的最新研究进展进行综述。

## Abstract (en)

Patients with advanced non-small cell lung cancer (NSCLC) carrying epidermal growth factor receptor (*EGFR*) activating mutations benefit from EGFR-tyrosine kinase inhibitor (TKI) treatment, however, most of TKI-treated patients eventually suffer drug resistant after 10-month treatments. Previous studies demonstrated that T790M mutation in exon 20 of EGFR gene would be the essential factor leading to EGFR-TKI resistance, leaving the mechanisms of which elucidative. Current research identified that T790M is an independent, favorable prognostic factor for predicting survival, but whether it is also a predictive biomarker for EGFR-TKI efficacy is still controversial. Up to date, techniques to detect T790M mutation in lung cancer have been greatly improved and the new therapeutic strategies emerged as well. In this review, we summarized the newly updated data about T790M mutation in terms of its mechanisms involved in EGFR-TKI resistant, clinical value, advanced detection assays and ongoing strategies against the mutation subtype.

近年来，非小细胞肺癌（non-small cell lung cancer, NSCLC）治疗领域里程碑式的改变是针对表皮生长因子受体（epidermal growth factor receptor, *EGFR*）基因突变阳性的晚期患者采用EGFR-酪氨酸激酶抑制剂（tyrosine kinase inhibitor, TKI）进行治疗。治疗获益的患者表现为无疾病进展期（progression-free survival, PFS）延长，客观反映率（objective response rate, ORR）提高，生活质量得到极大改善。尽管对初始EGFR-TKI治疗应答良好，但大部分患者在平均治疗10个月后会出现对此类药物的耐药。早期的研究^[1, 2]^发现，使用EGFR-TKI治疗后进展的肿瘤中，50%存在*EGFR*基因20号外显子第790位点的突变，即T790M基因突变。转染携带T790M基因突变的质粒的肿瘤细胞对EGFR-TKI的耐药性明显增加。随后更多的研究^[3]^结果也验证了肿瘤组织中T790M基因突变是导致EGFR-TKI耐药最主要的机制。随着临床研究的拓展和检测技术的更新，研究者发现使用EGFR-TKI前的NSCLC中也存在一定比例的*de novo* T790M基因突变^[4, 5]^，由此引发了T790M基因突变是获得性模式还是选择性模式的争论。此外，T790M基因突变是否与药物疗效预测和生存预后相关以及是否是EGFR-TKI的禁忌症等问题都还没有定论。本文总结近年来有关T790M和EGFR-TKI耐药方面的重要研究结果，阐述对T790M基因突变的共识和争议之处。

## 1 T790M基因突变的发现及耐药机制

### 1.1 T790M基因突变的发现

2005年Kobayashi等^[1]^对1例71岁、男性、应用EGFR-TKI治疗后疾病进展的NSCLC患者进行二次活检时发现，该肿瘤组织中除了治疗前检测出来的EGFR第19号外显子缺失之外（19Del），还增加了20号外显子T790M基因突变。该患者既往接受过二线吉非替尼治疗，达到完全缓解（complete response, CR）28个月后出现疾病进展。研究人员将T790M基因突变质粒转染肿瘤细胞后，该肿瘤细胞开始对吉非替尼产生耐药。同年Pao等^[2]^对6例接受TKI治疗后疾病进展的肿瘤组织/胸水标本再次进行*EGFR*基因突变检测，相比治疗前，3例标本（50%）新增加了T790M基因突变。随后对155例治疗前肿瘤标本的检测结果证实所有标本都不存在*de novo* T790M基因突变。体外试验证实同时携带*EGFR*基因21号外显子L858R基因突变和T790M基因突变的肿瘤细胞对TKI的耐药性是单独携带L858R基因突变肿瘤细胞的100倍。基于以上两个重要的研究结果，T790M基因突变被一致认为是导致EGFR-TKI继发性耐药的主要原因，它也是第一个被发现的与耐药相关的基因。随后更多的报道证明，不同种族或不同地域EGFR-TKI耐药患者的肿瘤组织中都发现有新增加的T790M基因突变。Chen等^[6]^采用ARMS法检测29例中国人NSCLC（86%为腺癌）后发现，二次活检肿瘤组织中T790M基因突变率为48%；Kosaka等^[7-9]^报道日本患者T790M基因突变率为50%-70%；Costa等^[10, 11]^报道美国患者T790M基因突变率为44%-86%。

### 1.2 T790M基因突变导致耐药的可能机制

Lynch等在2004年发现，NSCLC患者酪氨酸激酶区域存在*EGFR*敏感突变（19Del和L858R），EGFR-TKI可阻断三磷酸腺苷（adenosinetriphosphate, ATP）结合到细胞内酪氨酸激酶结构域，因此抑制受体自身磷酸化并导致下游的信号转导受阻，引起受体内化障碍。随后大量的论文和综述报道了EGFR-TKI对NSCLC治疗的疗效。2009年IPASS临床试验证实*EGFR*突变的患者可以从EGFR-TKI治疗中获益，尽管相比标准化疗，EGFR-TKI治疗患者的中位总生存期没有明显延长，但客观反应率提高，PFS延长，患者生活质量明显改善。随后F-SIGNAL、WJTOG3405、NEJGSG002、OPTIMAL和EURTAC等大规模、多中心、随机对照、前瞻性临床试验都证实了IPASS的结论^[12]^。

2007年Sharma等^[13]^详细分析了EGFR的药物敏感基因和药物耐药基因突变，前者主要包括19号外显子缺失（19Del）和21号外显子第858氨基酸位点突变（L858R），后者主要指20号外显子中第790氨基酸位点由苏氨酸（T）突变为甲硫氨酸（G），即T790M，基因水平表现为ACG突变为ATG。在亚裔、女性、不吸烟、腺癌患者中*EGFR*基因突变率高，19Del和L858R约占突变总数的90%，T790M约为2.5%。目前研究发现T790M突变总是与其它类型*EGFR*基因突变共存，主要是19Del或L858R，目前未见任何报道显示T790M突变单独存在于肺癌组织中。由于甲硫氨酸比苏氨酸空间占位大，因此形成空间位阻，改变了EGFR激酶区ATP的亲和性，导致EGFR-TKI类小分子药物不能有效阻断EGFR活化信号，从而失去对肿瘤细胞杀伤作用^[1, 2]^。

由于*EGFR*基因突变的患者采用EGFR-TKI治疗能获益，因此*EGFR*野生型患者不推荐使用EGFR-TKI治疗。在大多数针对T790M的研究中，入选患者也都是携带*EGFR*敏感突变并给与EGFR-TKI治疗的患者。对于*EGFR*野生型患者，由于不推荐进行EGFR-TKI治疗，因此EGFR-TKI是否会导致EGFR野生型的肿瘤发生T790M突变至今未知。

目前的共识是T790M基因突变是EGFR-TKI耐药的重要机制之一，但T790M的出现是从无（治疗前）到有（耐药后）还是从少（治疗前）到多（治疗后）至今还有争议。目前有两种假说，即获得性模式和选择性模式假说来解释此现象^[14, 15]^。获得性模式假说认为EGFR-TKI治疗前肿瘤细胞不存在*de novo* T790M基因突变，经过EGFR-TKI治疗后肿瘤新发生了T790M基因突变。选择性模式是指*de novo* T790M原本存在于极少数的肿瘤细胞中，当使用EGFR-TKI治疗时选择性清除了对药物敏感细胞后，耐药细胞即T790M基因突变细胞存活下来并发生增殖，因此这类突变细胞是经药物“选择”后出现的。支持获得性模式的证据主要依据是治疗前T790M基因突变率极低（0-2%），而耐药肿瘤中T790M基因突变率高^[3]^。此外，同一患者治疗前肿瘤中未检测到T790M基因突变，但治疗后疾病进展的肿瘤进行再次活检发现新增加了T790M基因突变^[2]^。支持选择性模式的证据是一些报道中采用敏感的检测方法进行T790M基因突变检测，发现EGFR-TKI-naive的肿瘤标本中*de novo* T790M突变并非罕见^[4, 5, 16-18]^，其突变率可高达79%。其中美国麻省总医院报道的外周血肿瘤细胞中T790M基因突变率为38%，这种无创性、可实时检测的标本为未来研究药物耐药机制及应对策略提供了宝贵的生物材料资源。

## 2 T790M基因突变检测方法

采用不同检测方法得到的T790M基因突变阳性率各不相同。文献报道的用于检测T790M基因突变的方法包括基因扩增后直接测序法（direct sequencing, DS）、突变富集PCR法、扩增阻碍突变系统（amplification refractory mutation system, ARMS）法、蝎形探针扩增阻滞突变系统（scorpions amplification refractory mutation system, SARM）法、基质辅助激光解析电离飞行时间质谱（matrix-assisted laser desorption/ionization time of flight mass spectrometry, MALDI-TOF MS）、PCR-集落杂交法（colony hybridization, CH）和下一代测序法（next-generation sequencing, NGS）等^[4, 19-22]^。在众多方法中，DS法的敏感性最低（表 1）。

**1:** 文献报道的T790M检测方法 Documented methods for T790M detection

Authors	Methods	T790 mutation rate (%)		Detection range (%)
		Pre-treatment	TKI-resistance	
Sequist and Bean *et al*^[11, 18]^	PCR-DS/qPCR	0-0.2	40-50	10-30
Chen *et al*^[6]^	ARMS	0	48	1
Taniguchi *et al*^[21]^	BEAMing	NA	43.5	0.1-1
Oh *et al*^[22]^	PNA-clamping PCR	8.2	NA	0.01
Kim *et al*^[19]^	Pyrosequencing	0.5	NA	10
Fujita *et al*^[4]^	PCR-CH	79	NA	NA
Su *et al*^[5]^	MALDI-TOF MS	25-32	83	0.4-2.2
PCR-DS: PCR-direct sequencing; qPCR: real-time PCR; ARMS: amplification refractory mutation system; BEAMing: beads, emulsion, amplification and magnetic; PNA-clamping PCR: peptide nucleic acid-clamping PCR; PCR-CH: PCR-clone hybridization; MALDI-TOF MS: matrix-assisted laser desorption/ionization time of flight mass spectrometry. TKI: tyrosine kinase inhibitor.				

采用更敏感的方法，可以提高治疗前标本（非肿瘤组织和肿瘤组织）的T790M基因突变阳性检出率。2008年Maheswaran等^[17]^利用CTC-chips从转移性肺癌患者的外周血中分离出循环肿瘤细胞（circulating tumor cells, CTCs）后，采用SARM法检测T790M基因突变，发现阳性率高达62.5%。采用MALDI-TOF MS法进行T790M检测，治疗前突变阳性率为25%-32%，该结果通过NGS进行了验证。此外，MALDI-TOF MS、克隆杂交法等除了对T790M突变进行定性检测，还可以对T790M基因突变进行半定量或定量检测。未来通过下一代测序技术不仅可以实现定性检测，也可以进行定量检测；不仅可以对手术标本检测，也可以对小标本、血液标本进行检测；还可以实现多基因检测。

## 3 T790M基因突变的临床相关因素

### 3.1 与疾病分期相关

日本的Inukai等^[16]^检测了280例患者，发现Ⅰ期/Ⅱ期患者T790M基因突变率为0.59%，而Ⅲ期/Ⅳ期患者T790M基因突变率为8.1%，提示T790M基因突变与疾病分期相关，晚期患者更多出现T790M基因突变。

### 3.2 与肿瘤发生部位相关

Oxnard等^[23]^对93例带有*EGFR*基因突变、经过EGFR-TKI治疗复发的NSCLC患者进行二次活检后发现，T790M基因突变的发生几率与取材部位相关，在肺与胸膜的肿瘤组织中常见，而在肝、骨、脑、肾上腺、子宫颈、皮肤和腹膜等部位的转移瘤中较少见；此外，T790M基因突变与性别、种族、吸烟状况、*EGFR*敏感基因突变类型、TKI药物种类及治疗时间无关。Rosell等^[24]^检测了EGFR-TKI治疗后疾病进展的129名患者，发现45例患者治疗前的肿瘤标本中存在T790M基因突变，该突变与骨转移及EGFR敏感突变类型相关，EGFR外显子19缺失患者多伴随T790M基因突变。

Arcila等^[25]^检测了99名对EGFR-TKI耐药的肺癌患者，发现脑脊液、肾脏、肾上腺、子宫颈、皮肤和胸壁的肿瘤中未检测到T790M基因突变。通过对14例标本的多处转移灶（2个-3个）分别进行突变检测，发现不同瘤灶间*EGFR*活性基因突变的一致率为100%，而T790M基因突变一致率仅为57%，提示T790M基因突变在不同部位具有更大的肿瘤异质性。

## 4 T790M基因突变是预测还是预后因素

### 4.1 是否与EGFR-TKI的疗效预测相关

2012年台湾Su等^[5]^采用MALDI-TOF MS检测了76名Ⅲb期/Ⅳ期NSCLC患者，发现*de novo* T790M基因突变率为31.5%。根据*EGFR*基因突变状态将患者分为3组，分别为L858R/19Del、L858R/19Del联合T790M基因突变和以上3种突变都不存在的患者。在56例经过EGFR-TKI治疗的患者中，具有*de novo* T790M基因突变的患者（*n*=23）对EGFR-TKI的应答时间明显短于不具有T790M基因突变（*n*=33）的患者，他们的PFS分别为6.7个月和10.2个月，经过EGFR-TKI治疗的两组患者的中位PFS都优于无EGFR活性突变的患者。尽管在总生存期（overall survival, OS）方面三组没有差别，但EGFR-TKI治疗前T790M阳性患者应用EGFR-TKI缓解时间更短，因此该研究认为*de novo* T790M基因突变是EGFR-TKI疗效的预测因子。Roselle等^[24]^也发现治疗前有T790M突变和无突变患者的PFS分别为12个月和18个月（*P*=0.05）。

Fujita等^[4]^则认为*de novo* T790M基因是否突变不预测EGFR-TKI治疗疗效，但突变丰度对疗效有预测作用。在该研究中作者采用克隆杂交的方式将T790M基因突变分为强阳性、中度阳性和无T790M基因突变组。结果显示，将患者按照治疗前肿瘤组织中是否存在T790M基因突变分组后，突变患者的中位至治疗失败时间（time to treatment failure, TTF）为9个月而无突变的患者是7个月，二者之间无统计学差异。将患者按突变丰度分组比较，T790M基因突变强阳性、中度阳性和无突变患者的TTF分别为41个月、7个月和7个月（*P*= 0.001, 9）。

其它一些小样本的报道则是探讨了疾病进展时T790M基因突变与EGFR-TKI疗效的关系。其中来自美国的Oxnard等^[26]^对22例Ⅰ期-Ⅲ期肺腺癌患者采用吉非替尼或厄洛替尼进行新辅助或术后辅助治疗，发现对EGFR-TKI治疗缓解时间长的NSCLC患者（TKI治疗结束后疾病发生进展者）T790M基因突变率低（0%），而缓解时间短的NSCLC（TKI治疗过程中疾病发生进展者）T790M基因突变率高达67%。荷兰的Becker等^[27]^观察了14例初始EGFR-TKI治疗后获得长期疾病控制的患者，这些患者经过标准化疗后再次发生疾病进展，后续采用EGFR-TKI治疗后，疗效达到部分缓解（partial response, PR）、疾病稳定（stable disease, SD）和疾病进展（progressive disease, PD）的分别为5例、7例和2例，其中T790M基因突变者分别为2个、1个和2个，这提示在初始EGFR-TKI治疗有效的患者中，再次使用EGFR-TKI治疗发生耐药的机制并不局限于T790M基因突变，可能有更多的耐药机制参与其中。

### 4.2 是否与患者生存相关

有关T790M基因突变与NSCLC预后相关性的研究结果较为一致。Oxnard等^[23]^发现58例EGFR-TKI治疗耐药后再次活检的患者中无基因突变患者相比携带T790M基因突变的患者疾病进展后的生存时间明显延长（19个月*vs* 12个月，*P*=0.036)。Uramoto等^[28]^观察了19例EGFR-TKI耐药患者生存情况，发现存在T790M基因突变者（*n*=8）的5年生存率为86.7%，而无T790M基因突变者5年生存率为13.3%。EGFR-TKI治疗后疾病进展的患者中，肿瘤中携带T790M突变者相对预后好的原因可能与多种因素相关：①携带T790M的*EGFR*基因突变细胞惰性生长；②无T790M基因突变的肿瘤中存在其它耐药机制（MET扩增等）、患者体能状态评分或新发病灶差异等其它临床因素的影响；③后续治疗药物的作用（T790M基因突变患者常采用EGFR-TKI联合化疗药物做为后续治疗，而该化疗药物倾向于对耐药肿瘤疗效更好）^[23]^。

另一项汇总6项临床试验的荟萃分析^[29]^显示，61例等待临床试验入组的EGFR突变的患者中14例在药物洗脱（TKI停药）期间发生疾病暴发（中位时间为8天）。研究发现与疾病暴发相关的因素包括EGFR-TKI治疗后至疾病进展时间和治疗前脑或胸膜存在转移，但与肿瘤组织中是否具有T790M基因突变无相关性，出现疾病暴发者与无疾病暴发者T790M基因突变率分别为55%和59%。该研究所纳入的患者并不是由于EGFR-TKI药物耐药后导致的疾病进展而是治疗中断导致的疾病进展，在这种特殊情况下疾病进展可能与T790M基因突变无关。

## 5 EGFR-TKI耐药的治疗策略

目前迫切需要解决的问题是，如何根据突变类型和突变丰度，在合适的时间选择适合的人群进行有针对性的治疗。Yang等^[30]^根据疾病控制时间、肿瘤负荷的改变和临床症状等因素将EGFR-TKI治疗失败患者的疾病进展临床模式分为快速进展、缓慢进展和局部进展，并根据不同的表现形式采取化疗、继续TKI治疗和TKI联合局部治疗等不同的治疗策略。此外，就分子机制而言，EGFR-TKI耐药是多种因素共同作用的结果，这些因素包括T790M基因突变、MET扩增、肝细胞生长因子（hepatocyte growth factor, HGF）上调、G蛋白偶联受体（G-protein-coupled receptor, GCPR）表达及小细胞肺癌转化等^[3, 9, 31-33]^。各种机制之间还存在交叉现象，使得EGFR-TKI耐药机制更加错综复杂，因此应根据主导耐药机制和临床因素，采取个体化治疗策略，克服EGFR-TKI耐药。

正在尝试中的治疗策略包括不可逆EGFR-TKI治疗、EGFR-TKI与EGFR抗体联合治疗、使用特异性T790M抑制剂和采用非特异性抑制剂等措施。

### 5.1 不可逆EGFR-TKI

T790M基因突变阻碍了EGFR与EGFR-TKI的结合或者增加了EGFR与其配体ATP的亲和力，最终无法阻断EGFR磷酸化所介导的信号转导而导致耐药^[34]^，因此不可逆性抑制EGFR可能克服继发耐药的产生。目前研究较多的有阿法替尼、Dacomitinib、Neratinib、XL647等。阿法替尼是EGFR和ErbB2的不可逆TKI，一项阿法替尼对比安慰剂治疗吉非替尼或厄洛替尼和一、二线化疗治疗失败NSCLC、随机、Ⅱb/Ⅲ期临床试验结果^[35]^显示，经独立评估后的阿法替尼组PFS为3.3个月，安慰剂组为1.1个月；ORR在阿法替尼组和安慰剂组分别为7%和1%；PFS和ORR在两组间均有统计学差异，但OS没有明显差异。Dacomitinib是一种不可逆抑制EGFR、HER2和HER4的TKI^[36]^。一项Dacomitinib治疗之前接受过≥1次化疗并且厄洛替尼治疗失败的NSCLC患者的Ⅱ期临床研究^[37]^显示，62例可评价的患者中，3例为PR，35例为SD。Neratinib是泛ErbB（EGFR、ErbB2和ErbB3）不可逆TKI，一项Ⅱ期临床试验共入组167例晚期NSCLC患者，均接受Neratinib口服治疗，其中A组为既往接受EGFR-TKI治疗且存在*EGFR*突变者，B组为既往接受EGFR-TKI治疗但无EGFR突变者，C组为既往未接受EGFR-TKI治疗的肺腺癌患者，三组患者的RR均为3%。XL647能不可逆性抑制EGFR、HER2、VEGFR-2和EphB4^[38]^，研究结果显示XL647能抑制L858R联合T790M基因突变的肿瘤生长。一项关于XL647的Ⅱ期临床试验^[39]^证明，在33例吉非替尼或厄洛替尼治疗缓解超过3个月后发生疾病进展的NSCLC患者中，12例（67%）存在T790M基因突变，用XL647（300 mg/d）治疗后，仅1例部分缓解，该患者不吸烟，EGFR外显子19缺失，血浆中无T790M基因突变；而T790M基因突变阳性的患者无一缓解，大部分在2个月内进展。综上所述，新一代的不可逆EGFR-TKI药物有效率仅2%-7%^[35, 37-39]^，与可逆EGFR-TKI相比并没有带来明显的临床获益。

### 5.2 EGFR抑制剂的联合

不可逆EGFR-TKI药物的疗效差强人意，如果同时给予针对EGFR包膜内的小分子抑制剂和包膜外抗体的联合治疗是否可以克服T790M耐药？2011年美国临床肿瘤学会（American Society of Clinical Oncology, ASCO）报道了阿法替尼联合西妥昔单抗治疗*EGFR*基因突变的吉非替尼或厄洛替尼治疗进展后的晚期NSCLC（2011 ASCO Annual Meeting, Abstract 7525）的结果，在39例存在T790M基因突变的患者中可评价疗效的有35例，其中33例（94%）疾病得到控制，有效率为51%（18/35）。18例T790M突变阴性的患者中可评价疗效的16例，其中94%疾病得到控制，有效率为56%。该结果显示，90%以上的EGFR-TKI耐药患者能够从阿法替尼联合西妥昔单抗治疗中获益，似乎为克服耐药提供一种较好的模式，但仍需等待最新结果公布及进一步研究证实。

### 5.3 TKI与MET抑制剂联合

2007年发现MET扩增是与EGFR-TKI获得性耐药相关的另一机制^[40]^。研究^[6, 8, 9, 11]^发现，EGFR-TKI耐药的NSCLC中大约有5%-33%同时存在T790M基因突变和MET扩增。同时使用EGFR和MET抑制剂可能会提高克服耐药NSCLC的疗效。Xu等^[41]^通过荷瘤小鼠模型（肿瘤细胞共表达T790M基因突变和MET扩增）证实单独抑制T790M基因突变或抑制MET扩增并不能使肿瘤明显缩小，而联合抑制二者可以使肿瘤明显缩小。目前研究中的针对MET扩增的抑制剂包括小分子抑制剂如ARQ197、SGX-523、XL-880、XL-184和抗MET抗体（如PF2341066、Ficlatuzumab和Rilotumumab）。Sequist等^[42]^研究显示，厄洛替尼联合ARQ197组患者的中位PFS为3.8个月，而厄洛替尼联合安慰剂组为2.6个月（危险比HR=0.81，*P*=0.24，校正HR=0.68，*P* < 0.05）。后续34例厄洛替尼治疗失败的患者交叉到厄洛替尼联合ARQ197治疗组，在23例可评价疗效的患者中出现2例PR，9例SD。2例PR患者均有MET扩增，说明EGFR-TKIs治疗失败的患者用MET小分子抑制剂是合理的选择。MetMAb是能特异性结合MET受体的单价单克隆抗体，2011年ASCO年会公布了OAM4558g的研究结果（2011 ASCO, Abstract 7505），MetMAb联合特罗凯可明显提高MET表达阳性NSCLC患者的PFS和OS，使这类患者死亡风险降低近3倍，可见TKI与MET抑制剂联合也是值得探讨的一种治疗模式。

### 5.4 T790M特异性TKI

当不可逆EGFR-TKI并没有达到预期的疗效时，研究者开始思索是不是直接抑制T790M基因突变可行。目前针对T790M基因突变特异性的抑制剂如CO-1686、AP26113和WZ4002正在进行临床前和Ⅰ期/Ⅱ期临床研究。CO-1686是一种口服、共价、小分子化合物，以剂量依赖的方式抑制肺癌*EGFR*基因突变细胞系H1975（EGFR L858R/T790M）和HCC827（EGFR 19DelE746-A750）的生长，目前处于Ⅰ期/Ⅱ期临床研究中；AP26113（NCT01449461）是一种合成的、口服的TKI，具有抑制表达L1196M（ALK）和T790M的NSCLC的活性，该药尚处于Ⅰ期/Ⅱ期临床研究中，已发现其主要副作用是疲劳、恶心、腹泻和肝毒性。WZ4002^[43]^苯胺酸骨架更适合T790M突变基因的空间构象，其与T790M突变型的亲和力是与野生型的100倍，其作用机制与抑制EGFR、蛋白激酶B（protein kinase B, AKT）和细胞外信号调节激酶（extracellular signal regulated kinase, ERK1/2）的磷酸化有关。WZ4002对含T790M基因突变的NSCLC细胞系有较高的抑制能力，在T790M基因突变肺癌鼠模型中也明显抑制肿瘤生长，该药目前仍处于临床前研究中。

### 5.5 其它针对旁路激活途径的药物

热休克蛋白90作为一种分子伴侣，有利于维持*EGFR*敏感突变的空间构像，应用热休克蛋白90抑制剂可以使EGFR降解。体内和体外研究^[44-46]^发现热休克蛋白90抑制剂可以使含T790M基因突变的NSCLC细胞系EGFR-PI3K-Akt-mTOR信号通路完全被抑制。目前热休克蛋白90抑制剂AUY922与厄洛替尼联合治疗获得性耐药NSCLC的Ⅰ期剂量爬坡研究正在进行（NCT01259089）。这方面的研究似乎向我们展示了克服T790M耐药的一种新的思路。

## 6 结语

综上所述，T790M基因突变是导致EGFR-TKI耐药的主要因素，但T790M基因突变确切耐药机制尚不清楚。目前T790M基因突变对预后有一定的判定作用，但对EGFR-TKI疗效的预测作用现有研究结果尚不一致。针对T790M基因突变的检测方法种类繁多，如何优化检测方法，提高检测的敏感性和特异性，帮助临床医生进行治疗决策，是需要深入探讨的课题之一。现有的临床实践仍然是根据临床特征进行耐药后治疗选择，随着T790M基因突变耐药分子机制的明确，如何根据分子发生机制的不同选择治疗方案将是未来的研究热点。
